# Prevalence and factors associated with sexual and reproductive health literacy among youth living with HIV in Uganda: a cross-sectional study

**DOI:** 10.1186/s12889-023-16399-9

**Published:** 2023-08-07

**Authors:** Benedicto Mugabi, Miisa Nanyingi, Richard Kabanda, Juliet Ndibazza, Peter Elyanu, John Baptist Asiimwe, Gorret Nazziwa, Gilbert Habaasa, Adeodata Kekitiinwa

**Affiliations:** 1 Uganda Martyrs University, PO Box 5498, Kampala, Uganda; 2 Baylor College of Medicine Children’s Foundation Uganda, PO Box 72052, Kampala, Uganda; 3 Aga Khan University, PO Box 8842, Kampala, Uganda; 4 Nakasero Hospital, PO Box 16595, Kampala, Uganda; 5 Population and Development Consult Limited, PO Box 23746, Kampala, Uganda

**Keywords:** Sexual and reproductive health, Sexual and reproductive health literacy, Youth, Human immunodeficiency virus, Prevalence, Associated factors, Clinical centre of excellence

## Abstract

**Background:**

Adequate sexual and reproductive health literacy (SRHL) among young people has been linked to informed sexual behaviours. Studies on SRHL have largely been conducted among the general adolescent population. Little is known about youth aged 15-24 years living with human immunodeficiency virus (YLHIV). There is a possible lack of SRHL in this population, considering the high rate of teenage pregnancies and unprotected sex reported by YLHIV. This study aimed to assess the prevalence and associated personal and environmental factors for SRHL among YLHIV at a high-volume urban HIV Clinic in Uganda.

**Methods:**

Through a cross-sectional survey, YLHIV receiving routine HIV care services at Baylor-Uganda HIV Clinic were interviewed using an adapted European Health Literacy Survey (HLS-EU). Using simple random sampling, eligible youth who received HIV care services between August and November 2019 were enrolled in the study. SRHL scores were computed using the HLS-EU index method; and individuals whose scores ranged from 34 to 50 were considered health literate. We used descriptive statistics to determine the prevalence. Potential associated personal and environmental factors (p<0.05) were identified by performing two-step inferential statistics, bivariate analysis and binary logistic regression. Odds ratios were calculated to estimate the likelihood of youth being health literate on sexual and reproductive health (SRH) issues in comparison with the reference categories, and 95% confidence intervals were determined to establish whether the relationships were statistically significant.

**Results:**

Of the 267 YLHIV interviewed at Baylor-Uganda HIV Clinic, 167 (62.5%) were female with a mean age of 18.9 years (SD± 2.8), and the majority (242; 90.6%) were vertically infected with HIV. Only 52 (19.5%) were health literate on SRH issues.

At the multivariate level, YLHIV who never had difficulty accessing SRH information were 0.391 times less likely to be health literate on SRH issues than their counterparts with challenges in accessing SRH information (Adjusted Odds Ratio [AOR] = 0.391, 95% CI =0.178 to 0.860; p= 0.019). YLHIV who did not find it easy to access SRH care service points were 2.929 times more likely to be literate in SRH than those who found it easy to access such services (Adjusted Odds Ratio [AOR] = 2.929, 95% CI =1.241 to 6.917; p=0.014). Additionally, YLHIV who did not listen to radio health talks were 2.406 times more likely to be health literate on SRH issues than those who did (AOR = 2.406, 95% CI =1.133 to 5.112; p=0.022).

**Conclusions:**

SRHL is an unmet need among YLHIV; only 19.5% were health literate on SRH issues. This could complicate the achievement of the UNAIDS sustainable development goal (SDG) of an HIV/AIDS-free generation by 2030 because low health literacy (HL) skills can affect the efficacy of almost all HIV disease prevention and health promotion efforts. Inaccessible SRH care service points and not listening to radio health talks were positively associated with SRHL, while having access to SRH information was negatively associated with SRHL.

## Background

The National Institutes of Health (NIH) has defined health literacy (HL) as the degree to which individuals can obtain, process, and understand basic health information and services needed to make appropriate health decisions [1]. HL represents the cognitive and social skills that determine the motivation and ability of individuals to gain access to, understand, and use information in ways that promote and maintain good health [2]. HL is not restricted to the ability to read and follow medical instructions. It includes a range of communicative and critical skills, such as searching for specific health information, evaluating information for credibility, balancing risks and benefits, expressing needs and negotiating preferences. It involves acquiring health information and knowledge about diseases, bodily conditions, and functions, which are evident determinants of health status and outcomes [3]. However, as information (learning to know) is only useful if reinforced by positive attitudes (learning to be) and useful skills (learning to do), the ability to recognize a potential problem must be accompanied by the will to identify it and the means necessary to avoid it [4]. “Life skills are abilities for adaptive and positive behaviour that enable individuals to deal effectively with the demands and challenges of everyday life” [5]. They include negotiating and exercising good judgement, maintaining self-esteem and handling pressure. Ensuring that individuals are health literate is desirable, as HL is assumed to be an asset that can reduce health disparities [6] and improve social capital [7]. Although it has been demonstrated that HL is one of the most important determinants of various aspects of public health [8], many researchers consider these life skills to be lacking in many populations worldwide, representing a challenge in the 21st century [9, 10]. Specifically, studies evaluating sexual and reproductive health literacy (SRHL) among youth living with HIV (a global chronic health problem) remain lacking despite young people contributing the largest share of the world’s population. In this study, youth are considered adolescents and individuals between the ages of 15 and 24 years.

There is a heightened awareness that sustaining sexual health in adolescence is essential to reproductive health and well-being in later life [11, 12]. Adolescence is conventionally understood as the time between the onset of puberty and the establishment of adult independence [12]. However, in many settings, young people are now reaching physical maturity (puberty) earlier, often engaging in sexual activity at a younger age and marrying later [12–14], with several societal and parental concerns regarding premature sexual activity, including unplanned pregnancy and sexually transmitted infections, among others [13].

Additionally, during adolescence, an individual acquires the physical, cognitive, emotional, social, and economic resources that are the foundation for later life health and well-being; the same resources are believed to define trajectories for the next generation [11]. Despite the global ambition of ending the AIDS epidemic as a public health threat for everyone by 2030, it is estimated that 1.3–2.2 million adolescents are living with HIV in Sub-Saharan Africa, both vertically and horizontally infected [10], and various studies have documented high rates of unprotected sex reported by youth living with HIV (YLHIV) even after infection (27–90%) [15]. While rates of unprotected sex among adolescents living with HIV are comparable to those among the general adolescent population [15], YLHIV are a key population for reducing HIV transmission to sexual partners and children given their reproductive potential.

Additionally, the high teenage pregnancy rate in Uganda [16] suggests that factors associated with high teenage pregnancy rates need to be explored among young people because they are sexually active, including those living with HIV. Additionally, studies have shown that among people living with HIV in sub-Saharan Africa, 35-75% desire to have children in the future due to increasing access to antiretroviral therapies [17]. These factors cannot be ignored since they could increase the HIV burden in society (especially in cases of poor viral suppression and risky sexual behaviours) and on personal health and quality of life. Furthermore, SRHL levels and associated factors have been investigated in the general population of young people [18, 19] but not specifically in the population of YLHIV.

The above findings in the literature suggest that if issues of SRHL are not investigated in this population, the United Nations’ efforts to end the AIDS epidemic as a public health threat for everyone by 2030 could be undermined because this knowledge gap puts society at risk of endless HIV infections. Thus, this study aimed to measure SRHL and understand factors associated with the observed outcome among YLHIV aged 15-24 years at an urban HIV clinic in Uganda.

Theoretical guidance on factors possibly associated with SRH literacy was largely provided by the sociocognitive model. This theory suggests that “human behaviour (learning) is determined by a continuous (reciprocal) interaction between cognitive (personal), behavioural, and environmental determinants” [20]. Learning a skill or information about a particular concept helps one to form an idea of how new behaviours are performed, and on later occasions, this coded information serves as a guide for action.

Social learning emphasizes the reciprocal relationship between social characteristics of the environment, how individuals perceive them, and how motivated and able a person is to reproduce behaviours they observe. People both influence and are influenced by the world around them.

**Fig. 1:**
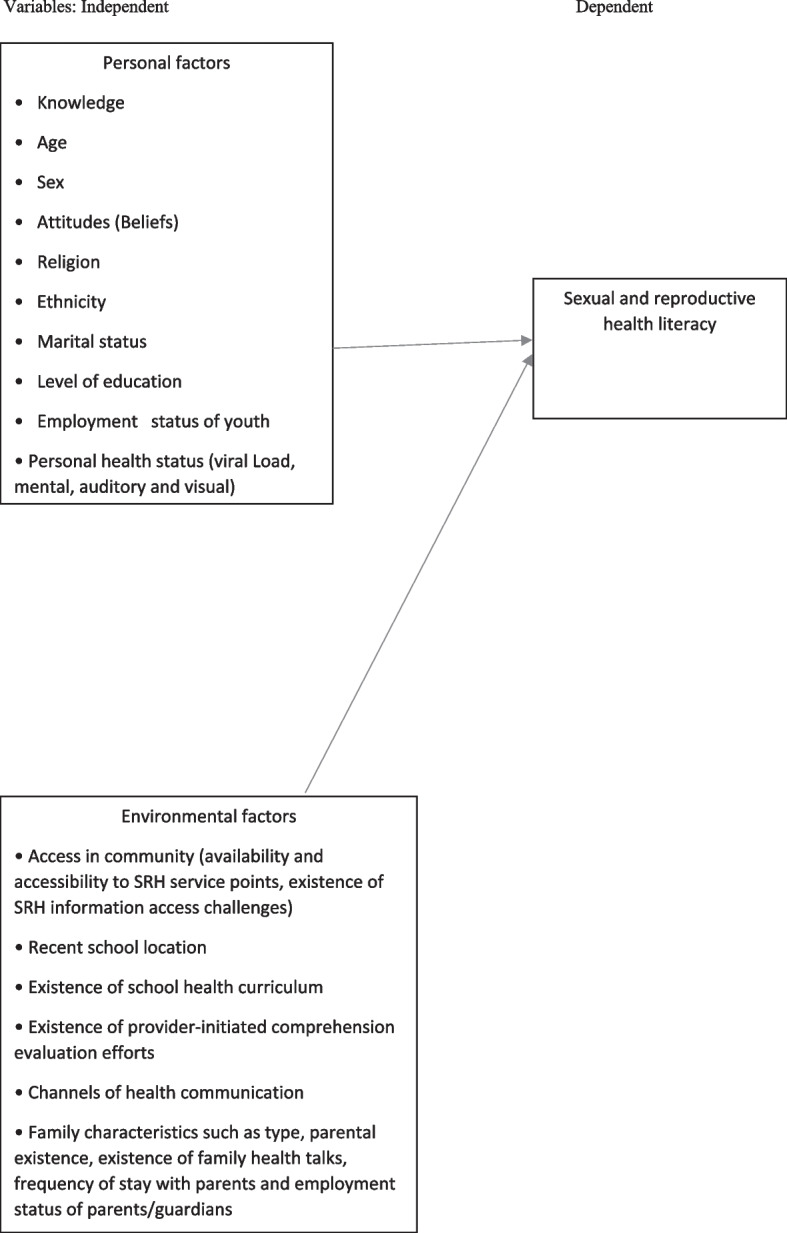
Theoretical framework illustrating the relationship between various factors and SRHL

Limited HL has multiple negative outcomes at the individual, societal and national levels. Thus, based on a literature review, several individual- and environmental-level factors have been shown to contribute to HL worldwide and thereafter to health outcomes [20, 21]. Figure 1 shows some of the selected personal and environmental factors assessed among the study population.

Knowledge gained from this study is projected to contribute positively to the design of targeted health interventions for YLHIV in a manner that reduces or even eliminates the risk of new HIV infections in their societies.

## Methods

### Study setting

Data collection was conducted at the Paediatric and Adolescent HIV Clinic of Baylor College of Medicine Children’s Foundation Uganda, in Kampala district, between August and November 2019. The clinic is a teaching and research institution providing educational opportunities for professionals at Makerere University College of Health Sciences and other healthcare training institutions. The clinic has five paediatric-adolescent service units (clinical care unit, nutrition unit, counselling unit, psychosocial services unit, play therapy and drama unit) and an estimated 4,517 YLHIV receiving HIV care and treatment services (clinical data, 2019). The Baylor-Uganda HIV Clinic was chosen as the site for this study because it is a clinical centre of excellence (CCE), has a high patient census, often with multicomplex medical, psychological and socioeconomic issues that require a higher level of care and attention, including the provision of intensive counselling sessions, health education, nutritional support, peer support activities and training opportunities in income-generating skills. Kampala district is a capital city in the central region of Uganda.

### Study design

This was a cross-sectional and analytical study involving a simple random sample of 267 YLHIV whose data were captured using a semistructured interviewer-administered questionnaire adopted and modified by the authors from the HLS-EU questionnaire to suit the study population.

The research assistants (RAs) were all Ugandan nationals residing in Kampala district with clinical research experience. The research assistants participated in five days of interactive training on all the study components, including a detailed review of the questions to ensure a shared understanding of the survey material. The RAs interviewed the 267 YLHIV in the youth’s usual clinic context (on their respective routine clinic visit appointment days). 

### Participants and recruitment

The following eligibility criteria for inclusion in the study were set to obtain the required sample: having been in care at the HIV clinic of Baylor College of Medicine Children’s Foundation Uganda for at least 12 months and having given written informed consent or assent to participate.

The reasons for excluding any potential participant included severe cognitive impairment whereby an individual is unable to answer interview questions and nondisclosure of one’s HIV sero status because of fear of accidental disclosure while interviewing youth on HIV-related content.

The study received approval from both local and international institutional review boards (IRBs). Additionally, permission was obtained from the Department of Research, Baylor College of Medicine Children’s Foundation Uganda. Written consent and assent were obtained from YLHIV prior to participation in this study.

### Sample Size Estimation

Using Yamane (1967) formula [22]: $\textrm{n} = \frac{\textrm{N}}{1+\textrm{Ne}^{2}}$

where

n = required sample size

e = error limit/level of precision (in this study, it is 5%)

N = the size of the target population (in this study, the prospective average monthly population of YLHIV with clinic appointments over 4 months from 15 July 2019 onwards was estimated at 606.75)

Placing information in the formula above at a 95% confidence level and an error limit of 5% revealed that the required sample size was 241 participants.

However, to account for possible nonresponse (estimated at 10%), the number of subjects was increased to 268 using the following formula: Final sample size = Effective sample size divided by (1- Nonresponse rate anticipated).

During data collection, one respondent did not answer 80% of the questions in each of the four questionnaire components of access, comprehension, appraisal and application of SRH information and hence was not included in the final analysis.

### Data collection procedure and sampling

A simple random sampling technique was used to select youth in this study. The simple random sample was first obtained in Microsoft Excel using the RAND function and sorting from the smallest to the largest randomly numbered participants from the initial Baylor-Uganda Electronic Medical Records (EMR) list of youth. The list obtained from the data department of Baylor-Uganda comprised only youth with clinic appointments falling in the planned data collection period (July to November 2019). During data collection, three trained research assistants (RAs) subsequently tracked and identified potential respondents from the waiting area using the final EMR-generated list of 268 randomly obtained samples of youth with appointment date information. The RAs then approached, greeted and briefed individual youth about the study procedure and significance to obtain their informed verbal and written assent or consent. Those who agreed to participate in the study were interviewed by the RAs in clinical examination rooms for an estimated average time of 45 minutes. To avoid interrupting patient care, youth were typically, although not always, targeted during tea and lunch breaks and after they had been seen through routine clinical procedures. The interviewer-administered semistructured questionnaire was developed based on a literature review and modified using the HLS-EU questionnaire to suit this study population.

The questionnaire had three parts: Part A (with 20 questions on sociodemographic/personal characteristics), Part B (with 26 questions on environmental factors presumed to be related to youth SRH literacy), and Part C (with 4 subsections on access, understanding, appraisal and application of SRH information with a total of 39 Likert-scale questions used to compute levels of SRH literacy). The personal characteristics included age, sex, ethnicity, religion, marital status and level of education of the youth. Other presumed factors included knowledge about SRH issues, attitudes and beliefs about SRH issues, such as belief in the possibility of an AIDS-free generation. Patient-specific factors included viral load status, visual status, auditory status, mental health status, and employment status. Environmental factors such as the existence of provider-initiated comprehension evaluation efforts, channels of health communication, school location, availability and accessibility to SRH service points, family characteristics such as type, parental existence, frequency of stay with parents, existence of family health talks, and employment status of parents/guardians were also considered.

The content in Part C focused on five selected SRH issues related to HIV transmission in this population, i.e., STI/STDs, contraception, pregnancy and childbirth, breastfeeding, and adherence to ART.

Two adolescent experts reviewed the questionnaire before data collection, and their input was incorporated into the final version. A pilot study was conducted comprising 20 YLHIV (10 boys and 10 girls) from another urban teenage health centre; the questionnaire was adjusted and finalized for use based on their responses and feedback. Thirty-nine (39) questions on the SRHL Part (Part C) were retained out of the initial 47 questions with minimal changes to the terminology used in the final version.

We used the Cronbach’s alpha coefficient reliability test to determine the reliability of the adjusted instrument using the SPSS program, as shown in Table 1. The average Cronbach’s alpha coefficient of the 39 SRH literacy items was 0.713, which is above the recommended threshold of 0.7 for a reliable research instrument [23, 24].

**Table 1:** The Cronbach’s Alpha Coefficients of Internal Consistency

Items Rated	Number of Items	Cronbach’s Alpha Coefficient
Accessing	9	0.712
Understanding	10	0.730
Appraising/Thinking	7	0.656
Applying	13	0.755
**Total**	**39**	**0.713**

### Analysis

The collected data were entered into and analysed using Statistical Package for Social Sciences (SPSS) Version 25. Of the 268 youth interviewed, one respondent did not answer 80% of the questions in each of the four subsections of access, comprehension, appraisal and application of SRH information and therefore was not included in the final analysis (n =267).

The SRH literacy scores were calculated based on the HLS-EU index using the following formula:

“Index-score = (mean–minimal value of mean) * (50/3)”, scaled by four levels: inadequate: 0–25, problematic: >25–33, sufficient: >33–42 and excellent: >42–50. Both the formula and the scales were adopted from the

HLS-EU-Q47 method [25]. Youth with sufficient and excellent scores constituted the SRH-literate portion, while those with inadequate and problematic scores constituted the SRH illiterate portion. Descriptive results were summarized as frequencies and percentages. We performed two-step inferential statistics to identify factors possibly associated with the SRHL level; bivariate analysis (Pearson chi-squared test was used to assess the association between the measured SRH literacy level, which was the primary outcome, and each of the various factors) and subsequent binary logistic regression to determine the potential factors ($p< 0.05$) with odds ratios to estimate the likelihood of youth being health literate on SRH issues in comparison with the reference categories. The 95% confidence interval was calculated to establish whether the relationships were statistically significant. We included all independent variables with $p< 0.05$ in the final model. No confounding factors were found or adjusted for during the analysis.

## Results

Participants

As shown in Table 2, 62.5% of the 267 youth interviewed were female, and the mean age was 18.9 years. Most were vertically infected with HIV (90.6%) and virally suppressed (77.5%). The majority had attended school (99.6%), were currently in secondary schools (64.7%), were in urban schools (80.8%), were Christians (76.4%), and their region of origin was the central part of Uganda (60.3%). The majority had one living parent (46.8%), lived in single-headed families (49.2%), and had one parent/guardian working (57.3%). Most of the parents/guardians were self-employed (67.7%).

**Table 2:** Sociodemographic/Personal Characteristics of Respondents

Sociodemographic characteristics ($n=267$)	Frequency	Percentage (%)
**Sex**		
Male	100	37.5
Female	167	62.5
**Last viral load in the past 12 months**		
Less than 1000 Copies/ml	207	77.5
More than 1000 Copies/ml	60	22.5
**Religion**		
Christian	204	76.4
Muslim	63	23.6
**Region of origin**		
Central	161	60.3
Western	55	20.6
Eastern	26	9.7
Northern	14	5.2
Southern	3	1.1
Non-Ugandan	8	3.0
**Age**		
15-19 Years	167	62.5
20-24 Years	100	37.5
**Mean age**		18.9
**Median age**		19.0
**Standard deviation**		2.8
**Occupation**		
Employed	75	28.1
Unemployed (Student)	168	62.9
Unemployed (Nonstudent)	24	9.0
**Ever attended school**		
Ever attended school	266	99.6
Never attended school	1	0.4
**Highest level of education attained**		
Primary	43	16.1
Secondary	172	64.4
Higher	51	19.1
Not Applicable	1	0.4
**Most recent school location**		
Urban	215	80.5
Rural	51	19.1
Not Applicable	1	0.4
**Youth living with**		
Both parents	48	18.0
One parent	117	43.8
A guardian	79	29.6
Marital partner	12	4.5
Alone	11	4.1
**Type of family**		
Single headed	133	49.8
Nuclear	118	44.2
Polygamous	13	4.9
Stays in orphanage	3	1.1
**Guardians/parents working**		
Both parents/guardians work	82	30.7
Only one parent/guardian works	155	58.1
None of the parents/guardians work	27	10.1
Not applicable; parents/guardians deceased	3	1.1
**Nature of work for guardians/parents**		
Self-employment	161	60.3
Informal employment	32	12.0
Formal employment	44	16.5
Not applicable	30	11.2

Source: Primary data (2019)

### Proportion of YLHIV who are health literate on SRH issues

Table 3 shows that only 19.5% of the study participants were health literate on SRH issues after dichotomizing the SRH literacy levels in Table 4.

**Table 3:** Prevalence of SRHL in YLHIV

Primary outcome	Frequency	Percentage (%)
Health Illiterate	215	80.5
Health Literate	52	19.5
**Total**	**267**	**100**

**Table 4:** Index Scores on SRHL for YLHIV

Index score levels for SRHL	Frequency	Percentage (%)
Inadequate (0–25)	81	30.3
Problematic (>25–33)	134	50.2
Sufficient (>33–42)	40	15
Excellent (>42–50)	12	4.5
**Total**	**267**	**100**
Mean index score		28.3
Median index score		27.8
Standard deviation		6.9

Based on the scale of four SRHL levels, half of the sample (50.2%) had problematic SRHL, and slightly below 5% had excellent SRHL. The mean SRHL score was 28.3, and the median was 27.8

**Fig. 2:**
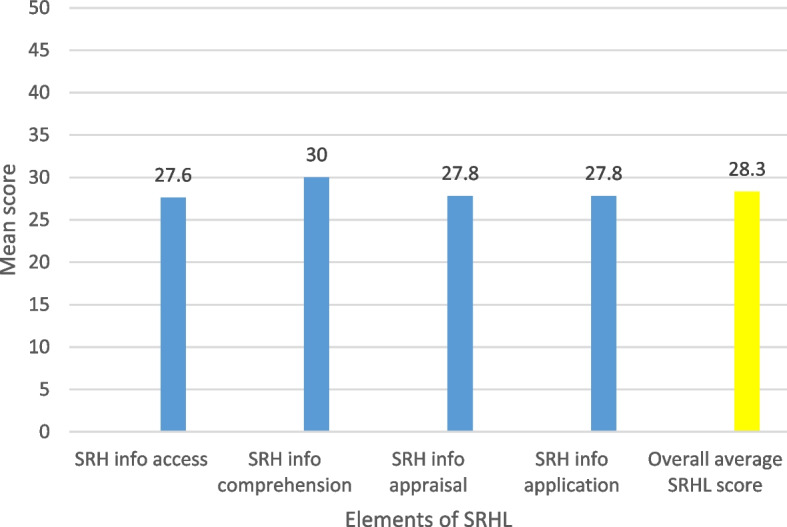
Mean SRHL scores among YLHIV

Figure 2 shows the mean score of the youth’s ability to access, understand, appraise and apply SRH information on a scale from 0-50.

### Factors associated with SRHL among YLHIV

Table 5 shows that at the bivariate analysis level, the youth’s religion ($\varvec{\chi }^{\textbf{2}}$ = 4.350, df = 1, p = 0.037) and age bracket ($\varvec{\chi }^{\textbf{2}}$ = 4.340, df = 1, p = 0.037) were significantly associated with their ability to access, understand, appraise and apply SRH information (i.e., SRHL).

**Table 5:** Bivariate Analyses between Personal Factors and SRHL among YLHIV

Variable	Category	Sexual and Reproductive Health Literate		Chi Sq. $({\chi }^{2})$	*P* value
		Yes (%)	No (%)		
Gender	Male	15 (15.0%)	85 (85.0%)	2.042 Df=1	0.153
	Female	37 (22.2%)	130 (77.8%)		
Marital status	Single	43 (18.5%)	189 (81.5%)	1.000 Df=1	0.317
	In union (Married)	9 (25.7%)	26 (74.3%)		
Last viral load in the last 12 months	Less than 1000 copies/ml	40 (19.3%)	167 (80.7%)	0.014 Df=1	0.907
	More than 1000 copies/ml	12 (20.0%)	48 (80.0%)		
Religion	Christian	34 (16.7%)	170 (83.3%)	4.350 Df=1	0.037*
	Muslim	18 (28.6%)	45 (71.4%)		
Age category	15-19 Years	26 (15.6%)	141 (84.4%)	4.340 Df=1	0.037*
	20-24 Years	26 (26.0%)	74 (74.0%)		
Region of origin	Central	27 (16.8%)	134 (83.2%)	9.708 Df=5	0.084
	Western	12 (21.8%)	43 (78.2%)		
	Eastern	6 (23.1%)	20 (76.9%)		
	Northern	5 (35.7%)	9 (64.3%)		
	Southern	2 (66.7%)	1 (33.3%)		
	Non-Ugandan	0 (0.0%)	8 (100.0%)		
Occupation	Employed	17 (22.7%)	58 (77.3%)	2.769 Df=2	0.250
	Unemployed (Student)	28 (16.7%)	140 (83.3%)		
	Unemployed (Nonstudent)	7 (29.2%)	17 (70.8%)		
Highest level of education attained	Primary	11 (25.6)	32(74.4)	4.898 Df=3	0.179
	Secondary	27 (15.7)	145 (84.3)		
	Higher	14 (27.5)	37 (72.5)		
	Not applicable	0	1 (100.0)		
Belief in HIV-free generation	Agree	44 (20.6%)	170 (79.4%)	1.945 Df=2	0.378
	Disagree	4 (11.1%)	32 (88.9%)		
	Neutral	4 (23.5%)	13 (76.5%)		
General awareness on SRH issues	Sufficiently aware	36 (20.0%)	144 (80.0%)	0.072 Df=1	0.788
	Insufficient awareness	16 (18.6%)	70 (81.4%)		
Mental health problems	Yes	9 (15.0%)	51 (85.0%)	1.019 Df=1	0.313
	No	43 (20.9%)	163 (79.1%)		
Difficulty in hearing	Yes	6 (24.0%)	19 (76.0%)	0.348 Df=1	0.555
	No	46 (19.1%)	195 (80.9%)		
Difficulty in seeing	Yes	11 (22.9%)	37 (77.1%)	0.442 Df=1	0.506
	No	41 (18.7%)	178 (81.3%)		

$\varvec{\chi }^{\textbf{2}}$= Pearson chi-square, Df= degree of freedom, p=probability value, ^*^statistically significant at the 0.05 level (2-tailed)

Source: primary data (2019)

**Table 6:** Bivariate Analyses between Environmental Factors and SRH Literacy among YLHIV

Variable	Category	Sexual and Reproductive Health Literate		Chi Sq. $({\chi }^{2})$	*P* value
		Yes (%)	No (%)		
Parental life status	Both parents alive	17 (17.3%)	81 (82.7%)	0.602 Df=2	0.740
	One parent alive	25 (20.0%)	100 (80.0%)		
	Neither parent alive	10 (22.7%)	34 (77.3%)		
Youth living with	Both parents	9 (18.8%)	39 (81.3%)	2.009 Df=4	0.734
	One parent	19 (16.2%)	98 (83.8%)		
	Guardian	18 (22.8%)	61 (77.2%)		
	Marital partner	3 (25.0%)	9 (75.0%)		
	Living alone	3 (27.3%)	8 (72.7%)		
Guardians/parents employment status	Both work	13 (15.9)	69(84.1)	1.318 Df=3	0.725
	Only one works	32 (20.6)	123 (79.4)		
	Neither of them work	6 (22.2)	21 (77.8)		
	Not applicable/deceased	1 (33.3)	2 (66.7)		
Nature of work of guardians/parents	Self-employment	26(16.1)	135(83.9)	4.276 Df=3	0.233
	Informal employment	6(18.8)	26(81.3)		
	Formal employment	13(29.5)	31(70.5)		
	Not applicable	7(23.3)	23(76.7)		
Type of family where the youth stays	Single-headed	25 (18.8)	108 (81.2)	0.514 Df=3	0.916
	Nuclear	23 (19.5)	95(80.5)		
	Polygamous	3 (23.1)	10 (76.9)		
	Orphanage	1 (33.3)	2 (66.7)		
Frequency of stay with parents	Everyday	20 (20.4%)	78 (79.6%)	0.919 Df=3	0.821
	At least once a week	2 (18.2%)	9 (81.8%)		
	Less than once a week	4 (28.6%)	10 (71.4%)		
	Not at all	26 (18.3%)	116 (81.7%)		
Read health articles in newspapers or magazines	Yes	32 (18.5%)	141 (81.5%)	0.300 Df=1	0.584
	No	20 (21.3%)	74 (78.7%)		
Listen to radio health talks	Yes	39 (24.5%)	120 (75.5%)	6.399 Df=1	0.011*
	No	13 (12.0%)	95 (88.0%)		
Watch television health talk shows	Yes	40 (20.4%)	156 (79.6%)	0.350 Df=1	0.554
	No	12 (17.1%)	58 (82.9%)		
Access the internet	Yes	36 (20.1%)	143 (79.9%)	0.140 Df=1	0.708
	No	16 (18.2%)	72 (81.8%)		
Existence of a health curriculum at the school	Yes	33 (19.8%)	134 (80.2%)	0.013 Df=1	0.910
	No	19 (19.2%)	80 (80.8%)		
Most recent school location	Urban	45 (20.9)	170 (79.1)	1.607 Df=2	0.448
	Rural	7 (13.7)	44 (86.3)		
	Not applicable	0	1 (100.0)		
Existence of youth-friendly SRH services at Baylor-Uganda	Agree	46 (19.7%)	187 (80.3%)	0.594 Df=2	0.743
	Disagree	2 (12.5%)	14 (87.5%)		
	Neutral	4 (22.2%)	14 (77.8%)		
Accessibility to SRH care service points	Easy to find	43 (25.9%)	123 (74.1%)	11.338 Df=1	0.001*
	Not easy to find	9 (9.0%)	91 (91.0%)		
Existence of family health talks	Agree	38 (23.6%)	123 (76.4%)	4.410 Df=2	0.110
	Disagree	10 (12.5%)	70 (87.5%)		
	Neutral	4 (16.0%)	21 (84.0%)		
Existence of provider-initiated comprehension evaluation efforts	Agree	42 (25.3%)	124 (74.7%)	11.672 Df=2	0.003*
	Disagree	4 (6.2%)	61 (93.8%)		
	Neutral	5 (14.3%)	30 (85.7%)		
Have SRH information access challenges	Yes	13 (10.5%)	111 (89.5%)	11.936 Df=1	0.001*
	No	39 (27.3%)	104 (72.7%)		
Primary source of help in case of SRH Issues	Parents	14 (12.1%)	102 (87.9%)	21.107 Df=9	0.012*
	Health workers	27 (35.5%)	49 (64.5%)		
	Fellow peers	7 (22.6%)	24 (77.4%)		
	Academic teacher	0 (0.0%)	6 (100.0%)		
	Internet	2 (12.5%)	14 (87.5%)		
	TV health programmes	1 (12.5%)	7 (87.5%)		
	Radio health programmes	0 (0.0%)	3 (100.0%)		
	Health articles in newspapers and magazines	0 (0.0%)	4 (100.0%)		
	Siblings	0 (0.0%)	2 (100.0%)		
	Guardian	1 (20.0%)	4 (80.0%)		

$\varvec{\chi }^{\textbf{2}}$ **= Pearson chi-square, Df= degree of freedom, p=probability value,** ^*^statistically significant at the 0.05 level (2-tailed).

Source: primary data (2019)

Table 6 shows that at the bivariate analysis level, listening to radio health talks ($\varvec{\chi }^{\textbf{2}}$ = 6.399, df = 1, p = 0.011), accessibility to SRH services and structures ($\varvec{\chi }^{\textbf{2}}$ = 11.338, df = 1, p = 0.001), health workers who evaluated the youth’s comprehension of SRH issues at clinic visits ($\varvec{\chi }^{\textbf{2}}$ = 11.672, df = 2, p = 0.003), youth who had challenges accessing SRH information ($\varvec{\chi }^{\textbf{2}}$ =11.936, df = 1, p = 0.001), and the youth’s main source of SRH knowledge and support ($\varvec{\chi }^{\textbf{2}}$ = 21.107, df = 9, p = 0.012) were significantly associated with the youth’s ability to access, understand, appraise and apply SRH information (i.e., SRHL).

### Multivariate analysis of factors associated with SRHL among YLHIV

A binary logistic regression model was run on factors that emerged as statistically significant at the bivariate analysis level, and the findings are presented in Table 7.

**Table 7:** Binary logistic regression Analysis results on Factors Associated with SRHL among YLHIV

Variable	Category	Sexual and Reproductive Health Literate		Crude OR (95% CI)	Adjusted OR (95% CI)	*P* value
		Yes (%)	No (%)			
Religion	Christian (ref)	34 (16.7)	170 (83.3)	1.000	1.000	
	Muslim	18 (28.6)	45 (71.4)	0.500 (0.259-0.966)	0.484 (0.227-1.033)	0.061
Age Category	15-19 Years (ref)	26 (15.6)	141 (84.4)	1.000	1.000	
	20-24 Years	26 (26.0)	74 (74.0)	0.525 (0.285-0.968)	0.526 (0.526-0.259)	0.075
Listen to radio health talks	Yes (ref)	39 (24.5)	120 (75.5)	1.000	1.000	
	No	13 (12.0)	95 (88.0)	2.375 (1.200-4.702)	2.406 (1.133-5.112)	0.022*
Accessibility of SRH care service points	Easy to find (ref)	43 (25.9)	123 (74.1)	1.000	1.000	
	Not easy to find	9 (9.0)	91 (91.0)	3.535 (1.64-7.618)	2.929 (1.241-6.917)	0.014*
Existence of provider-initiated comprehension evaluation efforts	Agree (ref)	42 (25.3)	124 (74.7)	1.000	1.000	
	Disagree	4 (6.2)	61 (93.8)	0.861 (0.271-2.738)	0.434 (0.111-1.689)	0.229
	Neutral	5 (14.3)	30 (85.7)	0.500 (0.078-3.186)	0.520 (0.067-4.006)	0.530
Have SRH information access challenges	Yes (ref)	13 (10.5)	111 (89.5)	1.000	1.000	
	No	39 (27.3)	104 (72.7)	0.312 (0.158-0.618)	0.391 (0.178-0.860)	0.019*
Primary source of help in case of SRH Issues	Parents (ref)	14 (12.1)	102 (87.9)	1.000	1.000	
	Health workers	27 (35.5)	49 (64.5)	0.549 (0.057-5.269)	0.690 (0.061-7.769)	0.499
	Fellow peers	7 (22.6)	24 (77.4)	2.204 (0.234-20.726)	1.927 (0.176-21.075)	0.764
	Academic teacher	0 (0.0)	6 (100.0)	1.167 (0.112-2.202)	1.111 (0.089-13.856)	0.591
	Internet	2 (12.5)	14 (87.5)	0 (0.000-0.000)	0 (0.000-1.000)	0.935
	TV health programmes	1 (12.5)	7 (87.5)	0.571 (0.041-8.049)	0.458 (0.027-7.695)	0.999
	Radio health programmes	0 (0.0)	3 (100.0)	0.571 (0.028-1.849)	0.501 (0.020-12.683)	0.587
	Health articles in newspapers and magazines	0 (0.0)	4 (100.0)	0 (0.000-0.000)	0 (0.000-1.000)	0.675
	Siblings	0 (0.0)	2 (100.0)	0 (0.000-0.000)	0 (0.000-1.000)	0.999
	Guardian	1 (20.0)	4 (80.0)	0 (0.000-0.000)	0 (0.000-1.000)	0.999
**Dependent Variable:** SRH literate YLHIV; SRH Literate =1, Illiterate =0						

*Statistically significant at the 0.05 level (2-tailed); Ref-Reference Category

Source: primary data (2019)

Table 7 shows that at the multivariate analysis level, inaccessible SRH care service points (p = 0.014), not having SRH information access challenges (p=0.019) and not listening to radio health talks (p = 0.022) were statistically and strongly related to youth’s level of SRHL. YLHIV who did not find it easy to access SRH care service points were 2.929 times (adjusted odds ratio [AOR] = 2.929, 95% CI =1.241 to 6.917) more likely to be SRH literate than those who found such services easy to access (reference category). Additionally, YLHIV who never had SRH information access challenges were 0.391 times (adjusted odds ratio [AOR] = 0.391, 95% CI =0.178 to 0.860) less likely to be health literate on SRH issues than their counterparts who had such challenges (reference category). YLHIV who did not listen to radio health talks were 2.406 times (AOR = 2.406, 95% CI =1.133 to 5.112) more likely to be health literate on SRH issues than those who did (reference category).

## Discussion

### Proportion of YLHIV who are health literate on SRH issues

This study explored the sexual and reproductive knowledge and sexual behaviour of YLHIV at Baylor College of Medicine Children’s Foundation Uganda by measuring the prevalence of SRHL and associated factors. Few comparative studies exist in East and Sub-Saharan Africa; those that do exist are either over 10 years old or were mainly conducted in Asia. Data were collected using an adopted and modified questionnaire focusing on five selected SRH issues in this population to enable SRHL measurement. The results revealed that most YLHIV were SRH illiterate (80.5%) after dichotomizing the SRHL levels. However, the observed prevalence of SRH illiteracy (80.5%) is slightly lower than that in the general population (92.4%) [19]. Additionally, most of those who were SRH illiterate in this study had SRHL scores falling in the range of ‘problematic’, with a mean score of 28; the majority of those who were SRH illiterate in the general population had scores falling in the range of ‘inadequate’ (65.5%), although with a lower mean score (19.2/50) than that found in this study (28.3/50) was found in a comparative study [19]. The better mean SRHL scores in this study potentially arise from the larger age range of this particular youth sample, and this finding regarding the influence of age is consistent with the findings of other studies that HL is associated with age [26, 27].

Surprisingly, the mean HL for youth receiving close to regular services at a service point where information dissemination is easy was 28, which was below the desired score of>33. One would expect that youth receiving such information would translate into HL. Thus, this finding is inconsistent with findings in Japan, where a positive association was shown between HL and health information access [28, 29]. The discrepancy can be attributed to age differences and the differences in the sampling frame in these studies. For instance, Suka et al. (2015) conducted a study in six different health facilities, and together with the study by Shibuya et al. (2011), the mean age of the sample participants was higher than that of the current study participants [28, 29].

### Factors associated with SRHL among YLHIV

The associated factors for SRHL levels were assessed by bivariate data analysis, revealing that age, religion, the primary source of SRH information and support, accessibility to SRH care service points, not listening to radio health talks, the existence of challenges in accessing SRH information and the existence of clinician-initiated information comprehension and validation efforts were statistically associated with SRHL at the 0.05 level of significance. The association between age and HL found in this study is consistent with the findings of other studies in China and Germany [30, 31]. Additionally, the association between HL and the primary source of SRH information and support found in this study is consistent with the findings in other studies where for instance, young persons attributed their HL scores to their perceived parental health orientation and the trust they had in health service providers [32, 33]. Furthermore, the association between religion and HL observed in this study is consistent with findings in other studies where religious participation and higher levels of religiosity were found to be positively associated with HL [34, 35].

However, at the multivariate analysis level, only three factors, i.e., inaccessible SRH care service points, not having SRH information access challenges and not listening to radio health talks, were strongly associated with SRHL. On the issue of whether an individual found it easy or difficult to access SRH care service points, youth living with HIV who did not find it easy to access SRH care service points were 2.929 times more likely to be SRH literate than those who found it easy to access such services (Reference Category). Surprisingly, one would expect that youth who find it easy to access SRH care service points could optimally seek and utilize readily available health services, including health education, counselling services and adequate access to medical logistics and supplies, which could translate into HL. However, this finding is consistent with findings from other studies that found that low HL is associated with greater use of health care services [36, 37].

Additionally, this finding appears to suggest the existence of complacent behaviours and laxity among youth who perceive or feel they can always access whatever SRH care service points they would wish to or that there is just a lack of intrinsic motivation among youth to seek and assimilate services readily available to them and to visit structures they perceive to be accessible to them frequently. This is a helpful and timely revelation for HIV programmers and strategists because it partially explains why certain HIV prevention and health promotion efforts, such as facility-based SRH service delivery, might fail to yield good outcomes. However, this could make other strategies of case identification, such as demand creation for SRH services among youth through community outreach and innovative, differentiated service delivery models, a success. Additionally, because this finding pinpoints the potential for a delay in achieving the first 95 of the UNAIDS ambitious targets intended to end the AIDS epidemic as a public health threat for everyone by 2030, stakeholders in the HIV programming and research work must devise innovative and strategic ways to counsel and reach out to youth of reproductive potential. In particular, measures to improve an individual’s self-efficacy should be encouraged, including verbal persuasion during interpersonal contact at any health service point of engagement.

There was also a significant relationship between the observed SRHL and the inexistence of SRH information access challenges in that individuals who never had SRH information access challenges were 0.391 times less likely to be health literate on SRH issues than their counterparts who had such challenges (Reference Category). This is also a helpful and timely revelation for HIV programmers and strategists since the sources of information accessed by youth might not be authentic or well-tailored to guide their proper decision-making. Another explanation might be that there could be information overload and mismatch of the SRH information shared such that it is meant for ordinary youth and not considerate of special and complex populations, including YLHIV. Additionally, SRH information might be skewed to reasonably guide youth in appropriate decision-making.

Alternatively, those who had SRH information access challenges might have applied the little information they acquired or had access to other unpopular sources of information and learning. There were also uncertainties regarding the family characteristics of this group.

Unexpectedly, not listening to radio health talks was found to be associated with HL. The YLHIV who did not listen to radio health talks were 2.406 times more likely to be health literate on SRH issues than those who did (Reference Category). Possible explanations could be that youth tend to obtain SRH information and support from either their parents, health workers or fellow peers, outside media influences, which is consistent with the social cognitive theory (SCT) used in psychology, education and communication and with previous reports in the literature specifically concerning the positive influence of peers on the SRHL levels of young people [19]. Thus, given the stigma and fear related to SRH in general, this finding appears to suggest the need for health programming to emphasize innovative and interactive interpersonal approaches to health communication to youth to improve HL and health outcomes because youth tend to share their health experiences with people they trust will keep their secret and not judge them.

Remaining health illiterate despite listening to radio health talks could be attributable to the incomprehensive and inadequate SRH information packages shared through public radio talk shows given the sensitivity of SRH topics. Thus, the information shared might be insufficiently practical or unique to meet the SRH needs of individual young listeners. Individuals might also remain health illiterate due to a failure or reluctance of the young listeners to put into practice the teachings conveyed through the talk shows.

### Limitations

In this study, possible limitations that might be useful for further research include that the sample might not exactly represent the nation demographically. For example, males were slightly underrepresented in this study. However, this finding is similar to that observed in the LAO People’s Democratic Republic [19].

Additionally, the findings might not be generalizable since data collection was performed using an adapted and modified tool and because of the special study setting (a clinical centre of excellence).

Additionally, since data were sometimes collected during working hours when the youth were supposed to be receiving routine clinical care, their concentration during the interview could have been interrupted, which could have compromised the quality of the data collected because of the extended stay in the clinic.

Culturally and religiously (in Christianity and Islam), premarital sex is prohibited in Ugandan society and could result in being stigmatized, punished, judged, rejected and becoming pregnant at an early age; thus, respondents might have given socially desirable answers to the questions related to sexual experience, resulting in an underestimation of their level of sexual activity.

Finally, although discussing sex is usually a cause for shyness and a source of possible prejudice in this age group [19], this was not a major concern in this study because the youth were interviewed in clinical rooms on a one-on-one basis to ensure privacy. However, several youth were concerned about whether their information would be shared with the health facility administration, fearing that they could be reprimanded or even denied services at the clinic. They might not have been fully reassured that the administration would not see and act on their answers. Thus, we cannot rule out that despite these measures, youth still felt insufficiently ‘safe’ to answer these questions truthfully. This could potentially result in the underreporting of sexual experience.

## Conclusions

SRHL is an unmet need for YLHIV, as only 19.5% of YLHIV are health literate on SRH issues. An overwhelming number of YLHIV (80.5%) who are sexually and reproductively illiterate are sexually active. This could complicate the achievement of the UNAIDS sustainable development goal (SDG) to reach an HIV/AIDS-free generation by 2030, especially if expedited action and investment are not promptly implemented to address this challenge. Low HL skills can affect the efficacy of HIV disease prevention and health promotion efforts.

An inability to access SRH care service points and not listening to radio health talks were positively associated with SRHL, while not having SRH information access challenges was negatively associated with SRHL.

The dynamics of listening to the radio in the context of the socioeconomic situation of youth, the scope of SRH information disseminated through radio, the way and time it is passed on to the audience and the extent to which it addresses the SRH needs of youth require further investigation.

### Implications for public health policy and practice

HIV infections among this study population have existed as a public health problem for a long time, and despite the substantial level of funding invested in the several programs designed to address the HIV burden, including Elimination of Mother to Child Transmission (EMTCT), the challenge of horizontal HIV transmission remains. Thus, the findings of this study offer insight into inadequate SRHL among youth living with HIV. This awareness will result in a deeper examination and synthesis of all possible drivers of the HIV burden beyond what is already known, leading to tailored interventions and the possible attainment of the UNAIDS goal to end the AIDS epidemic by 2030.

The findings, therefore, have multiple implications, such as emphasizing the need to swiftly scale up and sustain good quality, curriculum-based sexual education programs in schools through the newly developed National Sexual Education Framework (2018) with more focus on competencies, such as good HL, because previous interventions have proven that such programs can positively impact behaviours and significantly delay the initiation of sexual activity and increase contraceptive use [38, 39].

Libraries of authentic SRH information should be created and popularized, and youth should be guided on where these trusted pieces of information could be accessed through strategic and innovative means.

Devising innovative ways to periodically engage and attract youth at the SRH service delivery point and reaching out to them personally in the communities in which they live will be critical.

SRH services require improvement, including emphasizing and documenting clinician-initiated SRH information comprehension evaluation efforts at all youth clinic appointments, ensuring regular SRHL monitoring and implementing sustainable sexual behavioural surveillance systems among youth living with HIV.

Policy-makers should design special training programs for parents aimed at empowering and building their capacity to handle and explain complex SRH issues/topics brought to their attention by their children at the family level since, according to the findings of this study, parents are the main source of SRH knowledge and support for youth.

Designing, sustaining and evaluating the impact of innovative national programs, such as youth-only sexual education camps during holidays and through which youth could be taught about a wide range of SRH issues/topics in a youth-friendly environment regarding freedom of expression, would be beneficial.

## Data Availability

The datasets used and/or analysed during the current study are available from the corresponding author upon reasonable request.

## Abbreviations

SRH: Sexual and reproductive health
SRHL: Sexual and reproductive health literacy
HIV: Human immunodeficiency virus
CCE: Clinical centre of excellence
EMR: Electronic medical records
ART: Antiretroviral therapy
SDG: Sustainable development goals
REF: Reference number
